# A robot arm-assisted acupuncture system with motion and force monitoring: establishment and validation

**DOI:** 10.3389/fbioe.2025.1563263

**Published:** 2025-08-06

**Authors:** Ying-Chen Li, Yi Yu, Xuan Qiao, Liu-Jie Ren, Jing-Wen Xu, Xin Wang, Wei Gu, Wei Yao

**Affiliations:** ^1^ Shanghai Key Laboratory of Acupuncture Mechanism and Acupoint Function, College of Biomedical Engineering, Fudan University, Shanghai, China; ^2^ College of Medical Instruments, Shanghai University of Medicine & Health Sciences, Shanghai, China; ^3^ Department of Facial Plastic Reconstructive Surgery, Eye and ENT Hospital of Fudan University, Shanghai, China; ^4^ Department of Traditional Chinese Medicine, Naval Medical University, Shanghai, China

**Keywords:** acupuncture robot system, motion and force monitoring, acupuncture analgesia, adjuvant arthritis, innovation in traditional medicine

## Abstract

**Objective:**

Traditional manual acupuncture faces challenges in quantifying stimulation parameters and preserving techniques consistency. This study presents a robot arm-assisted acupuncture system replicating manual manipulations with precise motion and force monitoring.

**Methods:**

System validation was conducted through a Laser Vibrometer to measure multiple frequency-amplitude combinations, followed by comparative analysis between robotic and manual acupuncture manipulations using both motion and force monitoring. Additionally, therapeutic efficacy was evaluated through an adjuvant arthritis rat model, comparing the analgesic effects between robotic and manual acupuncture at the Zusanli acupoint.

**Results:**

The Laser Vibrometer validation showed high precision, with mean absolute errors below 0.2 mm across all frequency-amplitude combinations. Comparative analysis demonstrated strong similarity between robotic and manual acupuncture in needle trajectories and force distributions, with Bhattacharyya coefficients of 0.9931 and 0.9976 for Lifting-Inserting and Mountain Burning manipulations. The robotic system achieved analgesic effects comparable to manual acupuncture, significantly improving pain thresholds over control groups.

**Conclusion:**

This work lays the groundwork for quantifiable, reproducible acupuncture therapy, driving the development of intelligent devices for standardized clinical applications.

## 1 Introduction

Acupuncture, a traditional Chinese medical therapy (TCM), has gained global recognition for its effectiveness (Li and Tan, 2013) in treating conditions such as rheumatoid arthritis (Chou and Chu, 2018), stroke rehabilitation (Shen et al., 2010; Chavez et al., 2017; Zhang et al., 2015), and pain management (Vickers et al., 2018; Zhao, 2008). However, despite its widespread clinical application, traditional manual acupuncture faces significant challenges in modern medical practice. On one hand, the stimulation parameters and therapeutic effects of manual acupuncture are difficult to quantify, limiting its reproducibility in clinical and experimental settings. On the other hand, preserving and transmitting the manipulation techniques of master acupuncturists remains a formidable task. In contrast, modern techniques such as electroacupuncture have gained popularity due to their standardization and ease of use (Liu et al., 2021). Nonetheless, the essence of manual acupuncture lies in its mechanical stimulation, where the needle delivers afferent mechanical signals to acupoint tissues through techniques like Lifting-Inserting (LIM) and Twisting (TWM). Clinical and experimental studies have confirmed that manual acupuncture’s efficacy is closely tied to parameters such as frequency, amplitude, depth, and duration (Chang et al., 2014; Kim et al., 2010). As a result, the ongoing international controversy over acupuncture’s effectiveness may largely stem from the under appreciation of these critical manipulation techniques (Yang et al., 2015).

In recent years, advancements in robotics have introduced new opportunities for manual acupuncture. By integrating modern engineering with traditional acupuncture, researchers are exploring robotic systems capable of high-precision acupuncture operations. These innovations enable standardized manipulation parameters for clinical and experimental treatments through intelligent healthcare systems, with the ultimate goal of ensuring therapeutic effectiveness while reducing labor demands. Wu and Zhang (2022) developed a hand-eye coordination control system for an acupuncture robot that achieved fixed acupoint positioning and needle insertion. However, post-insertion manipulation, a fundamental skill for acupuncturists, play a key role in achieving therapeutic effects. Notably, achieving “Deqi” (a specific sensation) during manipulation is essential for maximizing these effects (Liu et al., 2005). Langevin et al. (2001) suggested that the Deqi response involves a biomechanical component known as “needle grasp”. Thus, whether robotic acupuncture operations can achieve the Deqi effect as traditional acupuncture is still an important consideration. Yu et al. (2024) designed a bio-inspired acupuncture robot combined with a human-machine interaction system that can implement flexible needle manipulations similar to manual acupuncture. Su et al. (2019) developed a hand-eye-force coordination acupuncture robot that achieved force constraints during needle manipulation, though these constraints were mainly used to prevent the collision between the needle and bones rather than facilitating the implementation of manipulation. At present, these robotic arm-based acupuncture devices have seldom focused on the actual implementation effects compared to manual acupuncture. In other words, designing acupuncture robots with purely engineering approaches while ignoring mechanical feedback from manipulation techniques - similar to designing robots for object sorting - deviates from clinical efficacy requirements. This approach cannot fully simulate acupuncture manipulation techniques, nor can it contribute to the scientific explanation of acupuncture’s effects.

This study is designed with two primary objectives to address these limitations: (1) The integration of robotic arm control with force signal acquisition, focusing on real-time measurement and validation of needle displacement and needle-tissue mechanical signals during the robotic replication of manual acupuncture manipulation. (2) The comparison of manual and robotic acupuncture effectiveness through animal experiments to definitively verify the efficacy of robotic operations. This work aims to establish and validate a robot arm-assisted acupuncture system with motion and force monitoring foundations, through the quantification and precise reproduction of traditional acupuncture manipulations. A six-degree-of-freedom (6-DOF) robot arm will be used to simulate the kinematic characteristics of acupuncture of LIM and Mountain Burning manipulation (a renowned traditional therapeutic manipulation with heat sensation) (Li, 2008; Qu et al., 2023). Prior to this, the motion precision of the robotic arm will be verified to ensure its capability to execute complex acupuncture tasks. Moreover, an acupuncture manipulation motion acquisition module is established using computer vision and deep learning techniques to estimate the kinematic parameters of manual acupuncture manipulations and generate robot needling trajectories. Meanwhile, utilizing the previously developed real-time needle force monitoring unit (Ding et al., 2003), continuous monitoring of the mechanical information during the operation of the robot arm is possible, allowing for comparative analysis with the data obtained from manual acupuncture. Finally, experiments will be performed on adjuvant arthritis (AA) model rats using robot arm acupuncture to validate the analgesic effects of the system. This research not only provides a new perspective for integrating modern technology and traditional Chinese medicine but also provides systematic guidance for the development of acupuncture robots and lays a foundation for future AI-enabled intelligent acupuncture devices.

## 2 Materials and methods

### 2.1 Robot arm-assisted acupuncture system

**FIGURE 1:**
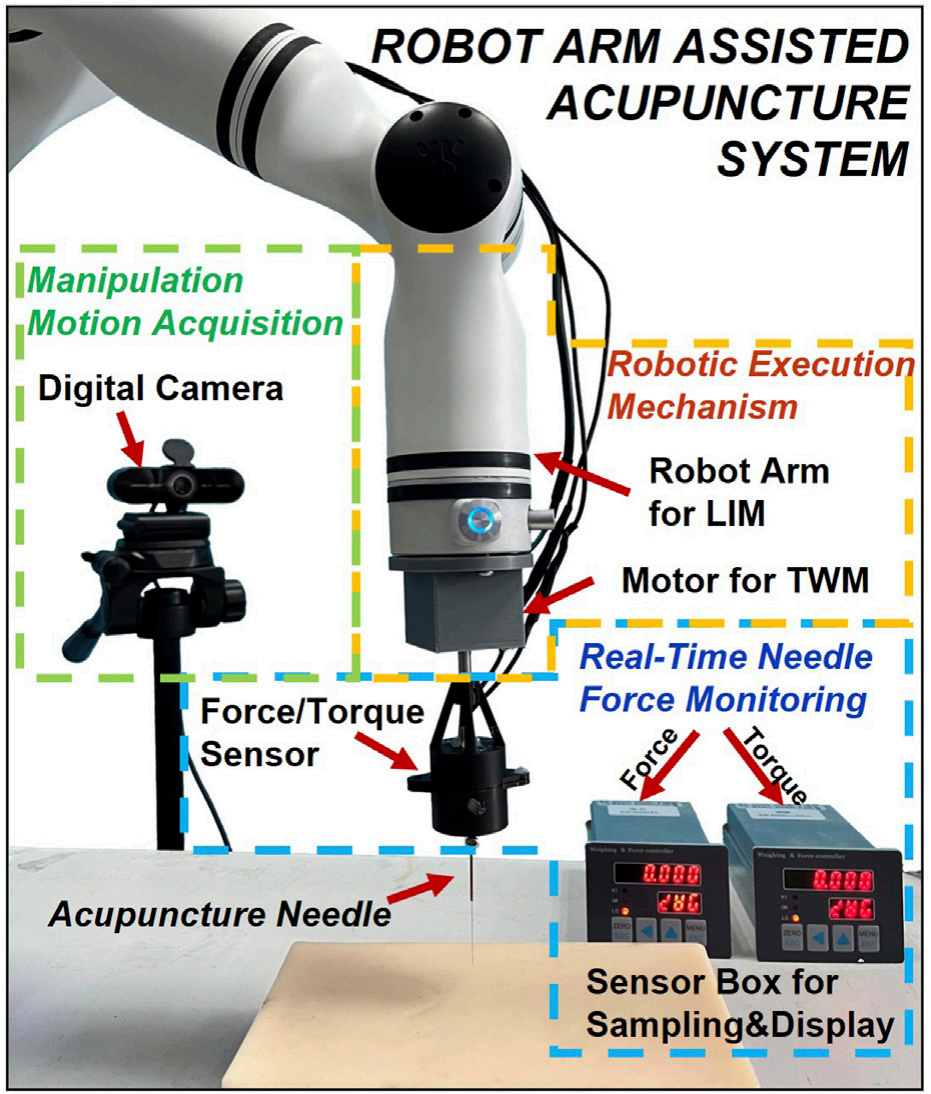
Architecture of the robot arm-assisted acupuncture system.

The architecture of the robot arm-assisted acupuncture system with motion and force monitoring functions is shown in Figure 1. The system is composed of three primary components: (1) an acupuncture manipulation motion acquisition module, (2) a real-time needle force monitoring unit, and (3) a robotic execution mechanism. During manual acupuncture, the acupuncture manipulation motion acquisition module and the real-time needle force monitoring unit are respectively employed to collect kinematic data and force/torque information. These data are subsequently analyzed and utilized to guide the robotic execution mechanism in performing precise acupuncture tasks.

Based on computer vision techniques, a digital camera captures high-resolution video of manual manipulations, which are processed frame-by-frame through deep learning algorithms to extract motion parameters. Furthermore, the real-time needle force monitoring unit developed by previous research (Zhang et al., 2008) can simultaneously measure vertical forces and rotational torques exerted by the needle on acupoints during acupuncture. These values of the needle force measurements potentially serve as objective indicators for distinguishing manipulations and observing certain physical aspects of the Deqi (Fan et al., 2024), although Deqi also includes subjective sensations reported by the patient. The robotic execution mechanism employs a robot arm (RM65, Realman, China) as its primary component for performing lifting-inserting manipulation (LIM). A rotational stepper motor (35CM015, China Leadshine Technology Co., Ltd, China) dedicated to twisting manipulation (TWM), and an additional force monitoring unit of the same type are secured to the flange plate interface at the end of the robot arm using 3D-printed connectors.

### 2.2 Verification of the robotic arm movement precision

Based on previous studies that identified optimal therapeutic parameters for manual acupuncture (Tong, 2018; Leow et al., 2018), the robot arm’s flexibility and accuracy were evaluated through LIM execution. The evaluation parameters comprised 16 combinations formed by pairing four frequencies (0.5, 1.0, 2.0, and 3.0 Hz) with four amplitudes (0.5, 1.0, 1.5, and 2.0 mm). The theoretical LIM motion curve (target) was set as a sinusoidal movement with a total duration of 40 s for each parameter combination. A Tukey (tapered cosine) window function was applied to smooth the initial and final 10 s of the signal, and the middle 20 s (10–30s) were used to observe the robot’s motion accuracy under stable conditions.

**FIGURE 2:**
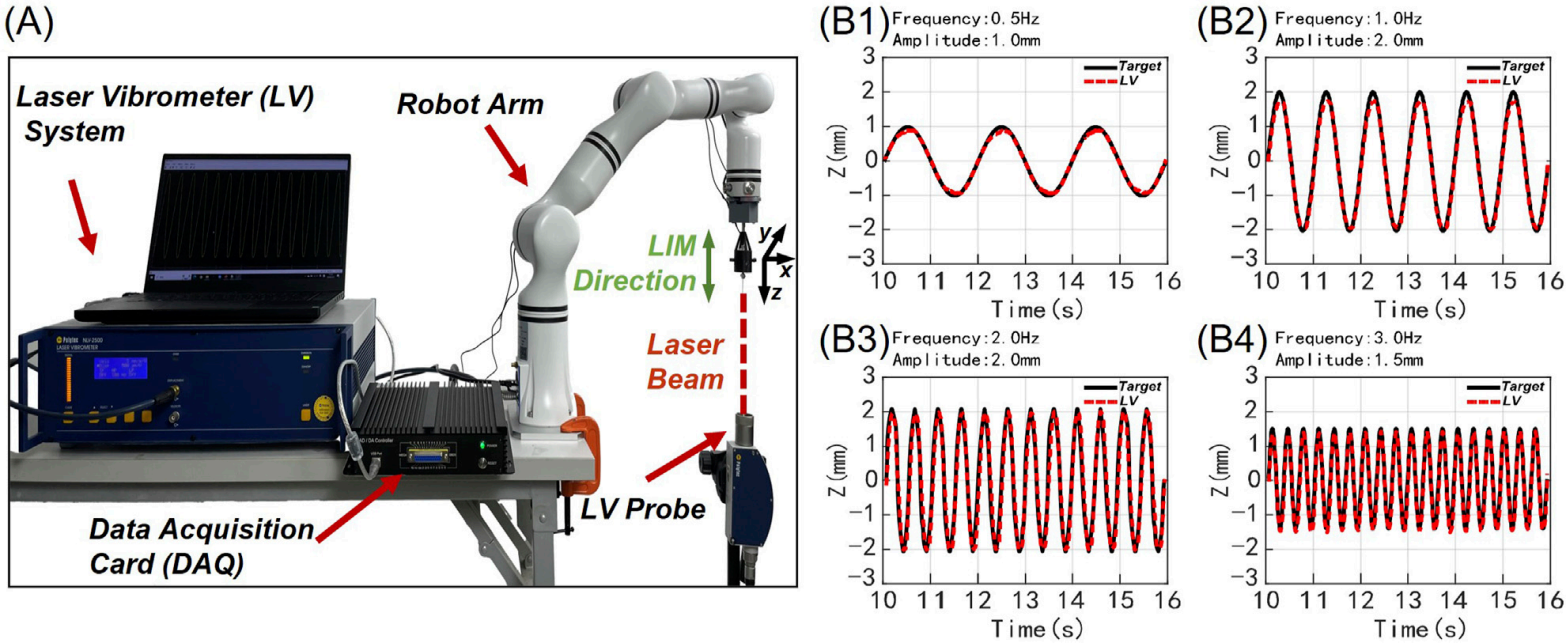
Verification of motion precision in robot arm: experimental diagram and partial results. **(A)** The LV detection system of the robot arm to record the acupuncture needle movement along the Z-axis. **(B1–B4)** Four experimental results of 16 frequency-amplitude combinations were displayed over a duration of 7 s. The red line represents the needle movement collected by the Lv system for robot arm acupuncture, while the black line indicates the pre-set theoretical target curve. (B1) 0.5 Hz frequency, 1.0 mm amplitude. (B2) 1.0 Hz frequency, 2.0 mm amplitude. (B3) 2.0 Hz frequency, 2.0 mm amplitude. (B4) 3.0 Hz frequency, 1.5 mm amplitude.

A Laser Vibrometer (LV-2500, Polytec, Germany) was employed to measure the displacement to verify the precise control of the robot arm, as shown in Figure 2A. The LV was fixed directly below the robot arm’s end-effector to capture its linear motion along the Z-axis, which represents the needling motion of the acupuncture. The LV outputs an analog voltage signal (500 μm/V) that was digitized using a data acquisition card (DAQExpress 2.0, National Instruments, United States) at a sampling frequency of 100 Hz. Partial experimental results for these manipulations are shown in Figures 2B1–B4. Additionally, the Mean Absolute Error (MAE) between the target and the measured needling parameters was calculated for all parameter groups, with results presented in Table 1.

**TABLE 1:** MAE and RE between the target values of the robotic arm and measurements.

Mean absolute error (MAE) (mm)/percentage-relative error (RE)		Amplitude (mm)			
		0.5	1	1.5	2
Frequency (Hz)	0.5	0.043/8.6%	0.047/4.7%	0.067/4.4%	0.075/3.7%
	1	0.054/10.8%	0.073/7.3%	0.080/5.3%	0.091/4.5%
	2	0.044/8.8%	0.077/7.7%	0.099/6.6%	0.118/5.9%
	3	0.077/15.4%	0.080/8.0%	0.110/7.3%	0.191/9.5%

### 2.3 Real-time needle force monitoring

The real-time needle force monitoring unit comprises a needle-holding force sensor coupled with its associated data sampling and display module. This sensor is designed to detect the mechanical load resisting needle movement during acupuncture, including linear force parallel to the longitudinal axis and torque opposing rotational manipulation. To meet these requirements, the sensor employs a hollow thin-walled cross-beam structure, with silicon strain gauges and piezoelectric ceramics used to measure the vertical forces and the rotational torques experienced by the needle, respectively. The acupuncture needle is mechanically fixed to the distal end of this sensing element while the practitioner grips the prosthesis handle fixed to the proximal end. Consequently, all loads applied by the practitioner to the prosthesis handle are transmitted through the sensing element to the acupuncture needle itself. Conventional strain gauges bonded to the sensing element can detect minute deformations resulting from applied forces and torques. Before use, acupuncture needles of various specifications are selected according to the target acupoints and mounted on the force sensor.

The measurement ranges of the force sensor are±4.8 × 10^−4^ N·m for torque and ±1.2N for axial force, with resolutions of 4.0 × 10^−6^ N·m and 1.0 × 10^−2^ N, respectively. After analog-to-digital conversion, the sensor acquires force measurement data at a sampling rate of 80 Hz, which is transmitted in real time to the host computer box and PC for visual display.

**FIGURE 3:**
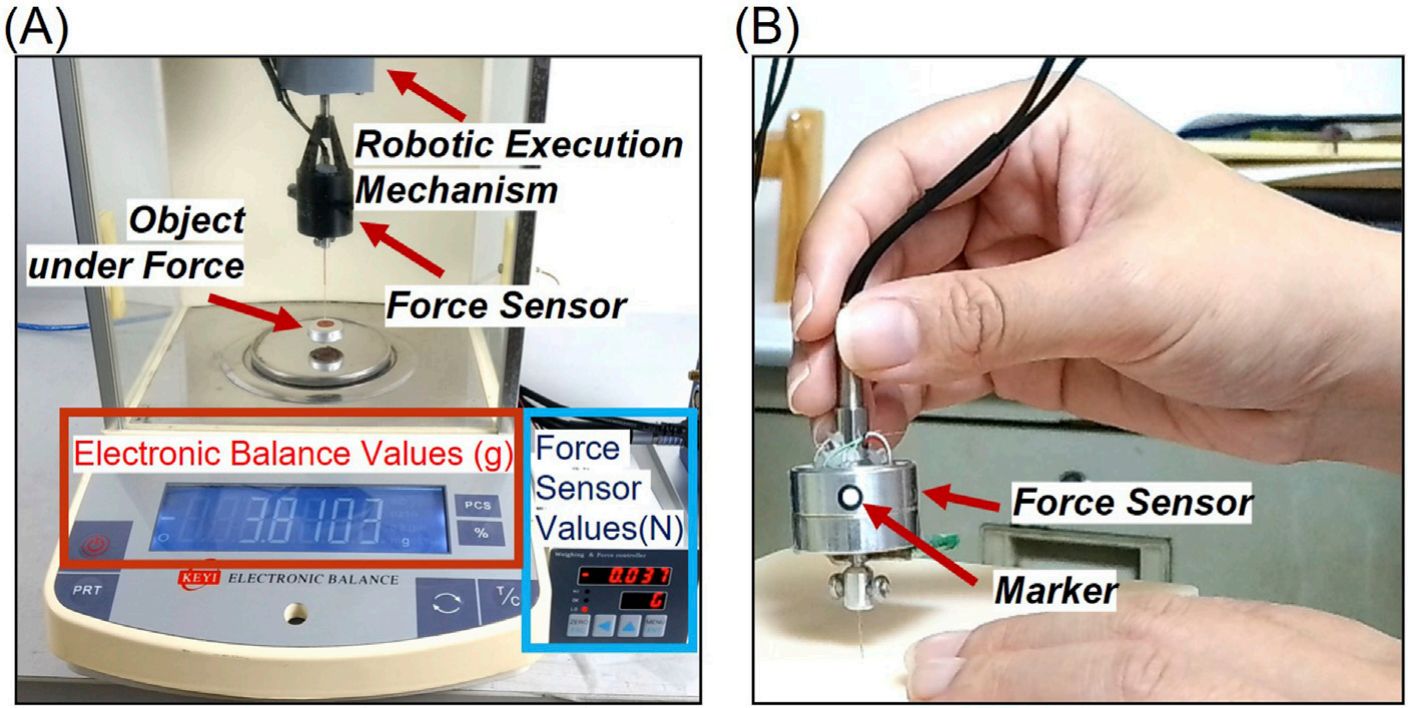
**(A)** Comparison of the difference between the force detected by the sensor and the actual force exerted on the acupoint tissue during needling. The force applied by the acupuncture needle to the object is measured and displayed by an electronic balance, while the reaction force exerted on the acupuncture needle by the object is collected and displayed via the force sensor. **(B)** Acupuncture manipulation motion acquisition. The motion information was extracted by tracking circular markers with a diameter of 3 mm, featuring a white interior and black outer ring.

To compare the difference between the force detected by the sensor and the actual force exerted on the acupoint tissue during needling, the force sensor was mounted on the robotic execution mechanism with the acupuncture needle securely gripped. The object placed stably on an electronic balance (FA1004, Changzhou Keyuan Electronic Instrument Co., Ltd., China, measurement range: 1.0 × 10^−4^–100g) was subjected to different forces as the robot arm was controlled to move downward or upward. Figure 3A illustrates the comparison between the force values obtained by the force sensor and the actual weight exerted by the acupuncture needle on the object.

### 2.4 Acupuncture manipulation motion acquisition

A high-definition digital camera (C60E, EMEET, China, 30 fps, 3840 × 2160 resolution) was employed to capture both manual acupuncture manipulations and their robotic replications. The motion information was extracted by tracking the black border, white-filled circular marker with a diameter of 3 mm, as illustrated in Figure 3B. Using a pre-trained deep learning model YOLO (v5.0, Ultralytics, United States), the marker in each video frame was detected. MATLAB (R2023a, The MathWorks, United States) was utilized to convert the pixel information of the markers into calibrated motion data for the entire needling process.

For manual manipulation acquisition, the marker was affixed to the side of the force sensor held by the practitioner, positioned parallel and directly facing the camera to minimize distortion. For robotic acupuncture, the marker was placed on the end-effector interface of the robotic arm. To mitigate potential image distortion due to perspective effects, two markers placed 6 cm apart in a straight line were used for displacement calibration.

### 2.5 From manual acupuncture manipulations to robotic replication

To comprehensively capture and enable the robotic arm to replicate the manipulation of manual acupuncture, an experienced acupuncturist conducted manual acupuncture utilizing both the acupuncture manipulation motion acquisition module and the real-time needle force monitoring unit. A silica gel skin model was selected as the recipient object for its stable mechanical properties and tactile similarity to human tissue, making it suitable for capturing consistent motion and force data during manipulation acquisition and robotic replication. The acupuncture needle was affixed to the front end of a force sensor, while the acupuncturist gripped the handle at the opposite end of the sensor. By manipulating the force sensor-needle assembly, the acupuncturist performed standard clinical LIM and Mountain Burning manipulation on the skin model. The acupuncture manipulation motion acquisition module recorded and the complete process of needle manipulation, from initial skin contact to skin penetration, manipulation application, and needle withdrawal. Meanwhile, the real-time needle force monitoring unit captured the forces exerted on the acupuncture needle within the skin model. The motion and force data in a time series were aligned, enabling the manual acupuncture manipulation to be analyzed in detail.

The kinematic data from manual acupuncture were processed through control algorithms to generate motion commands for robotic replication. An Inverse Kinematics algorithm was employed to calculate joint angles, enabling the robotic arm to follow the recorded trajectory. During robotic execution on the same skin model, both motion and force signals were again captured. Subsequently, these data were compared with those from manual acupuncture for analysis.

### 2.6 Analgesic effects of robotic acupuncture

#### 2.6.1 Animals and approval

This study was approved by the Professional Committee of Animal Protection of Shanghai University of Traditional Chinese Medicine (no. PZSHUTCM2401150001, date of approval: 30 December 2023). Healthy male Sprague - Dawley (SD) rats with a weight of 180 ± 20 g at the specific pathogen-free grade, were kept in the same standardized environment and provided with sufficient lighting, water, and food.

After the experimental procedures, animals were euthanized in accordance with the American Veterinary Medical Association (AVMA) Guidelines for the Euthanasia of Animals (2020). Each animal was placed individually into a 10 L euthanasia chamber, and carbon dioxide (CO_2_) was gradually introduced at a flow rate maintained at 30% of the chamber volume per minute to minimize distress and avoid nociceptor activation by carbonic acid prior to the onset of unconsciousness. To ensure the completeness of euthanasia, CO_2_ flow was continued for at least 1 min after respiratory arrest.

#### 2.6.2 Pain measurement

Rats were acclimated to the laboratory environment for 48 h prior to experimentation. On days 1 and 2, baseline thermal and mechanical pain thresholds were assessed. On day 2, an inflammatory pain model was induced via subcutaneous injection of Complete Freund’s Adjuvant (CFA, Beyotime Biotechnology, China) into the left hind paw. Day 3 was allocated to observe and maintain the animals without intervention, allowing the inflammatory response to fully develop. On day 4, upon verification of the established inflammatory pain model, baseline thresholds for both thermal hyperalgesia and mechanical allodynia were re-evaluated. Subsequently, acupuncture intervention was administered, followed by a post-treatment reassessment of thermal and mechanical nociceptive thresholds.

**FIGURE 4:**
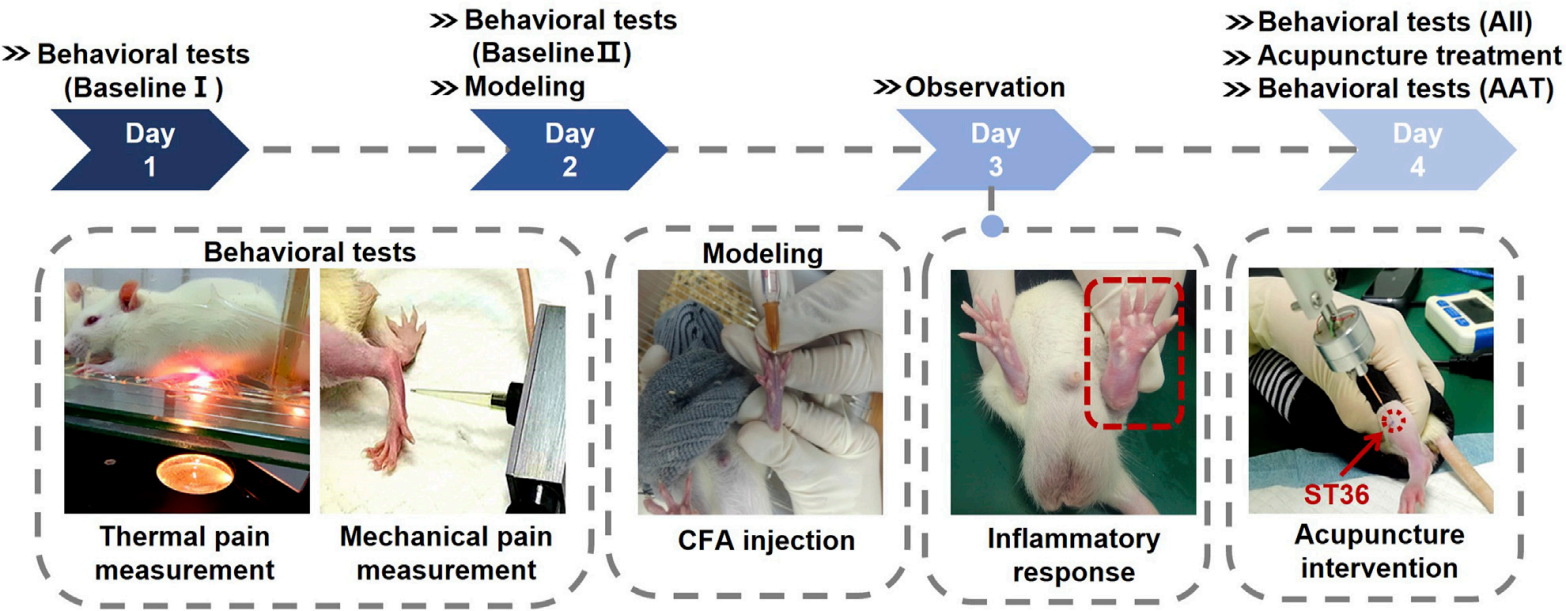
Protocol for the animal experiment. The rats were acclimated for more than 48 h before establishing the acute adjuvant arthritis (AA) model, and then the animals were acupunctured on Day 4. Pain threshold measurements were conducted on Day 1, Day 2 (before modeling), and Day 4 (before and after the acupuncture treatment).

The thermal pain measurement was performed in a quiet environment maintained at 25°C. The rats were placed in the cages of the thermal pain meter and allowed to move freely. After the rats had acclimatized to the environment for 20 min, the soles of their feet were irradiated with bright light by the IITC 336 GT PAW/TALL thermal pain meter (IITC Life Science, United States) (Figure 4). When the rats sensed pain, they exhibited a paw retraction response. The latency between the pain stimulus and paw retraction was recorded. Three measurements were taken at 5 min intervals, and the average value was used. The maximum stimulation time was set to 20 s to prevent injury to the rats.

To measure mechanical pain, the rats were restrained to expose their left legs, and then stimulated on the soles of their feet with an IITC Electric Von Frey (Ugo Basile, Italy) (Figure 4). The electronic pain meter recorded the instantaneous force value when the rats made a paw retraction response. The mechanical pain measurement was repeated three times, and the average of the three median values was taken as the mechanical pain threshold.

Since the basal pain threshold and the pain threshold after model induction differed for each group of rats, the pain measurement results after acupuncture treatment could not directly reflect the analgesic efficacy. To address this issue, the concept of pain threshold recovery rate (PTRR) was introduced, that is:

$$PTRR=\frac{AAT-AII}{B\text{ase}line-AII}$$

Where AAT indicates the pain measurement after acupuncture treatment (AAT), AII indicates the pain measurement after inflammation induction (AII), and Baseline indicates the results of pain threshold on days 1 and 2, which is considered as the basal pain threshold. When the value of PTRR is closer to 0, the analgesic effect is worse; when it is closer to 1, the analgesic effect is better.

#### 2.6.3 Experimental groupings and acupuncture treatment

A total of 24 rats were randomly allocated into four groups (n = 6 per group): control groups (CG), needle retention groups (NR), manual acupuncture groups (MA), and acupuncture robot groups (AR). After establishing the inflammatory pain model, group-specific interventions were applied. The control group received no acupuncture treatment. In the NR group, after needle insertion and manipulation to elicit Deqi, the needle was retained *in situ* for 10 min without further manipulation. The MA group received LIM treatment from a skilled acupuncturist (2Hz, ±1 mm, 10 min), While the AR group received the same LIM protocol executed by the robotic system.

Prior to the acupuncture intervention, the rats were immobilized and their left hind limbs were depilated. The Zusanli acupoint (ST36) was selected for its established analgesic efficacy (Dong and Ma, 2012). This acupoint is located on the posterolateral aspect of the knee joint, approximately 5 mm below the fibular head. For the acupuncture procedure, needles with a diameter of 0.3 mm were inserted to a depth of approximately 6 mm at the ST36 acupoint, and the needles were removed after 10 min of acupuncture manipulation. This standardized experimental design facilitated a systematic comparison of various acupuncture techniques, including the robotic approach, within the context of acupuncture-induced analgesia.

#### 2.6.4 Inflammatory pain model

Rats were immobilized, and the soles of their left hind paws were sterilized with alcohol swabs. A needle was then carefully inserted into the subcutaneous tissue between the second and third toes, followed by the slow administration of 0.2 mL of CFA. After the withdrawal of the needle, pressure was applied to the injection site for 1 minute to prevent leakage. Successful induction of the inflammatory pain model was characterized by significant erythema and edema in the injected paws (Figure 4). Additionally, a thermal pain threshold of less than 5 s and a mechanical pain threshold below 60 g were employed as criteria for confirming the establishment of the model.

#### 2.6.5 Statistical analysis

All data are presented as mean ± standard deviation (SD). The normality of the datasets was assessed using column statistics. For normally distributed data, differences between groups were analyzed by independent samples t-tests, followed by appropriate *post hoc* tests for multiple comparisons. For non-normally distributed data, non-parametric tests were employed. Each group consisted of six independent samples at the time of statistical analysis. Statistical analyses were conducted using GraphPad Prism 9 (9.0.0, GraphPad Software, United States), with a significance level of P < 0.05.

## 3 Results

### 3.1 Evaluation of motion accuracy for robotic arm

Figure 2B presents representative motion results of the robotic arm performing LIM for four selected parameter sets out of the 16 experimental combinations. The position shift of the needle at the robot’s end-effector demonstrates steady and smooth needling trajectories within the frequency range of 0.5–3 Hz (corresponding to 0.five to three lifting-inserting movements per second). Comparing the measured values with the target, the observed needling amplitudes and frequencies closely align with the predetermined experimental values. These results indicate that the robotic arm can effectively execute basic needling manipulation while maintaining stability and safety.

The Mean Absolute Error (MAE) and percentage-Relative Error (RE) calculations between the theoretical values of the robotic arm and the actual measurements from the Laser Vibrometer (LV) are listed in Table 1. The RE is calculated as the percentage ratio of MAE relative to the amplitude of the motion. This dimensionless parameter allows for better evaluation of the error under different needling manipulation parameters within the context of the same kinematic control algorithm. Throughout all 16 parameter combinations, the MAE remains below 0.2 mm. This demonstrates that the robot achieves high stability in satisfactory control of millimeter-scale needling movements. The lowest RE of 4.4\% was observed in the 0.5 Hz/1.5 mm group (MAE = 0.067 mm), while the highest RE of 15.4\% occurred in the 3 Hz/0.5 mm group (MAE = 0.077 mm). For groups with amplitudes greater than 0.5 mm and frequencies lower than 3 Hz, RE increased with frequency at constant amplitude, and decreased with amplitude at constant frequency. This indicates that the robot’s motion error increases with higher LIM frequencies and smaller amplitudes. Besides, 3 Hz frequency or 0.5 mm amplitude combinations showed relatively higher REs. These results demonstrate that within the range of clinical manual acupuncture parameters, the robot can execute precise needle movements with minimal error when following predefined clinical manipulation motion plans.

### 3.2 Real-time force monitoring of acupuncture needle

The electronic balance readings (in grams) were converted to force values (in Newtons) using the gravitational acceleration (9.8 N/kg). Prior to the experiment, the electronic balance was zeroed with the object placed on it. After determining the posture of the acupuncture robotic arm, the force sensor measured the reactive force exerted on the acupuncture needle by the object. The forces collected by the sensor demonstrated robustness. Compared to the actual force applied by the acupuncture needle to the object as measured by the electronic balance, the results from seven experimental groups showed an average error of 0.003N (0.0003g), as presented in Table 2. This indicates that the force obtained using the real-time needle force monitoring unit is essentially equivalent to the force applied by the acupuncture needle to the acupoint tissue. In group number 7, the object’s weight was equal to both the axial tensile force detected by the force sensor on the acupuncture needle and the measurement value of the electronic balance after the object lost contact with it. The error was 0N, confirming the accuracy of the real-time needle force monitoring unit’s measurements. The selected object weight was 3.81g.

**TABLE 2:** Specific experimental force data of sensor accuracy validation.

Number	Electronic balance readings force values (N)	Force sensor value ±SD (N)	Absolute error(N)
1	0.000	0.000 ± 0.000	0.000
2	0.085	0.085 ± 0.001	0.000
3	0.226	0.218 ± 0.003	0.008
4	0.299	0.297 ± 0.003	0.002
5	0.390	0.378 ± 0.005	0.012
6	0.550	0.551 ± 0.007	0.001
7	−0.037	−0.037 ± 0.001	0.000
MAE			0.003

Values are expressed as mean ± standard deviation (SD) based on five repeated measurements.

### 3.3 Motion and force of manual acupuncture

**FIGURE 5:**
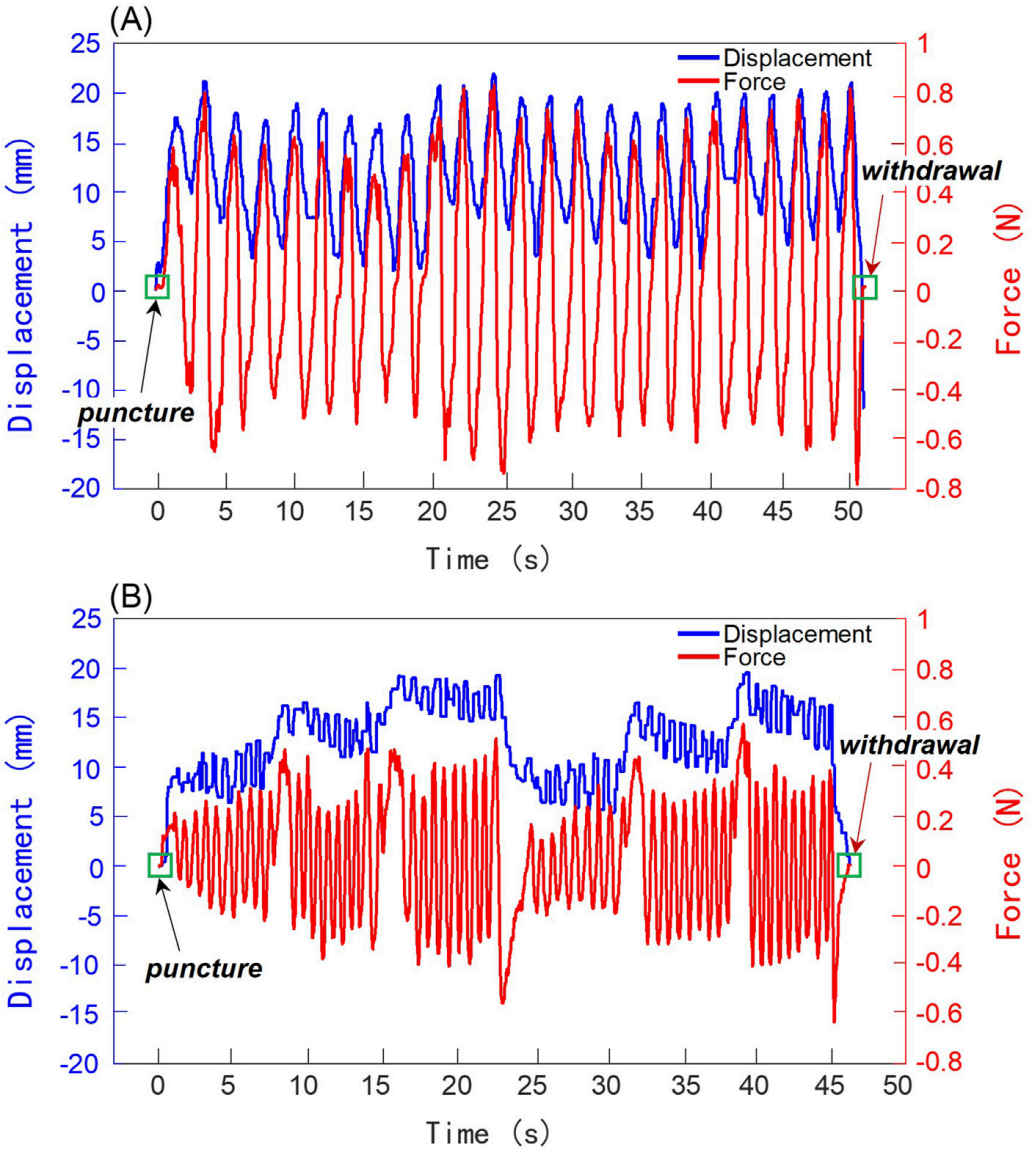
Results of manual acupuncture manipulations performed by a clinical acupuncturist. The left and right ordinate axes represent displacement and force, respectively. Blue curves indicate displacement, while red curves show force. **(A)** Lifting-Inserting Manipulation (LIM). **(B)** Mountain Burning manipulation.

As shown in Figure 5, the entire manual acupuncture process was recorded, capturing both needle displacement and the applied force. Since the recording of the acupuncture manipulations began as the needle tip approached the surface of the skin model, positive displacement values indicate the acupuncture needle’s penetration into the skin model. The magnitude of positive displacement represents the insertion depth, while negative values indicate the needle’s withdrawal from the skin model. Throughout the entire manual acupuncture process, the forces exerted on the acupuncture needle were comprehensively and instantaneously captured by the real-time needle force monitoring unit. The acquired force demonstrated consistency in periodicity and phase with the displacement, confirming the reliability of the real-time needle force monitoring unit. Positive force values represent the pressure experienced by the needle during insertion, while negative values indicate the pulling force during withdrawal.

During the initial puncture phase, the values of both displacement and force started from zero and increased with the depth of insertion. For the LIM, as shown in Figure 5A, the motion approximated a sinusoidal wave with a lifting-thrusting displacement amplitude of 7.5 mm and a frequency of 0.5 Hz. The force signal’s frequency matched this pattern, with force amplitude ranging from −0.8 N to +0.8 N. For the Mountain Burning manipulation, as illustrated in Figure 5B, the displacement revealed that the needle was first inserted to the upper 1/3 of the skin model’s depth (insertion depth of about 10 mm), where LIM was performed approximately nine times with an amplitude close to 2.5 mm. This was followed by insertion to the middle 1/3 (about 15 mm depth) and then to the lower 1/3 (about 20 mm depth), with similar LIM performed at each level. Subsequently, the needle was withdrawn from the lower 1/3 to the upper 1/3 of the acupoint depth. This Mountain Burning manipulation, consisting of a three-part insertion, was repeated twice. The frequency of the force signals generally aligned with the motion frequency, while the amplitude range varied according to the needle’s depth within the skin model, approximately ±0.2 N, ±0.3 N, and ±0.4 N for the respective depths. During the transitional insertion phases between segments, force values increased positively, ranging from 0.4 to 0.6 N. Conversely, during the withdrawal phases between repetition cycles, force values increased negatively, ranging from −0.4 to −0.6 N. For both the LIM and the Mountain Burning manipulation, following the therapeutic manipulation, the acupuncturist withdrew the needle from the skin model. At this point, the displacement and force values intersected at zero, marking the moment when the acupuncture needle completely exited the skin model. Furthermore, based on the characterization of the motion and force information, it is possible to distinguish acupuncture stimulation by needle puncture, therapeutic manipulation, and withdrawal.

### 3.4 Comparative analysis of manual and robotic replication acupuncture

#### 3.4.1 Comparison of kinematic and force between robotic and manual acupuncture manipulation

**FIGURE 6:**
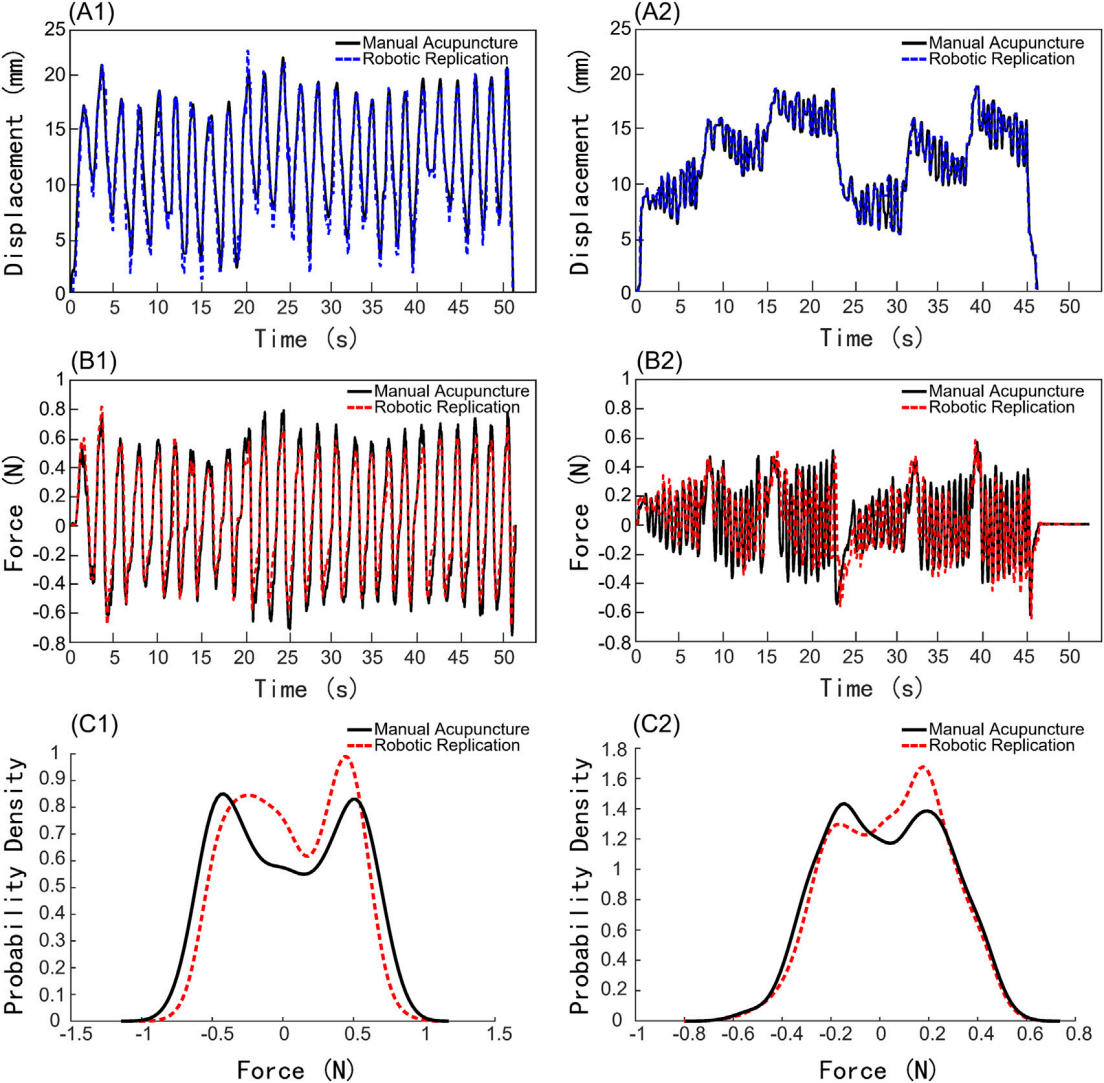
Comparison of kinematic and force results between robotic and manual acupuncture manipulations. **(A1–A2)** Displacement profiles. **(B1–B2)** Force profiles. **(C1–C2)** Force probability density distributions. (A1), (B1), (C1) Lifting-Inserting Manipulation (LIM). (A2), (B2), (C2) Mountain Burning manipulation.

As shown in Figures 6A1–A2, the motion waveform of the robotic replication almost perfectly overlaps with that of the manual manipulations. This indicates that the needle movement trajectory of the robotic arm acupuncture is extremely similar to that of manual acupuncture by an acupuncturist. Moreover, the force exerted on the needle during robotic arm acupuncture shows an overall trend similar to that of manual acupuncture, as illustrated in Figures 6B1–B2. Besides, the force probability density distributions of both manual acupuncture and robotic replication were analyzed, as shown in Figures 6C1–C2. The probability density distributions of forces generated by manual manipulation and robotic replication showed remarkable consistency. The overlapping distributions indicate that the robot successfully replicated the force characteristics of manual manipulation, maintaining similar force ranges and occurrence probabilities. The Bhattacharyya coefficients were 0.9931 and 0.9976 for the LIM and Mountain Burning manipulation, respectively. This high degree of similarity suggests that the robotic system effectively captured the essential force patterns of traditional manual acupuncture.

The comparative displacement graphs of manual and robotic replication clearly demonstrate that robotic acupuncture can mimic the clinical physician’s acupuncture manipulations with high precision, achieving almost complete consistency in needle movement. This demonstrates that the robotic arm can simulate manual acupuncture manipulations flexibly and accurately. The force comparison graph between manual and robotic acupuncture shows that under identical tissue conditions, when both methods apply the same manipulation, the forces exerted by the needle on the receiving tissue are fundamentally consistent. This suggests that, theoretically, consistent motion can achieve the same stimulation effects, indicating that acupuncture robots could significantly contribute to standardized treatment.

#### 3.4.2 Thermal and mechanical pain measurement

**FIGURE 7:**
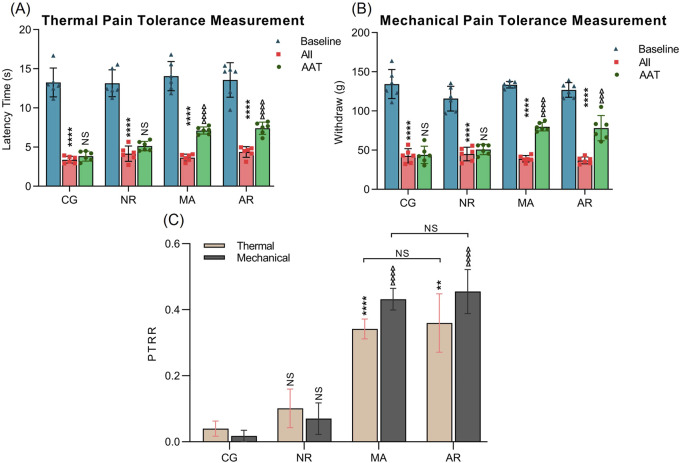
The results of thermal and mechanical pain tolerance measurements. **(A)** Thermal pain tolerance measurement. **(B)** Mechanical pain tolerance measurement. *P < 0.05, **P < 0.01, ***P < 0.001 and ****P < 0.0001 vs. Baseline pain threshold within the group; ∆ P < 0.05, ∆∆ P < 0.01, ∆∆∆ P < 0.001 and ∆∆∆∆ P < 0.001 vs. AII pain threshold within the group. **(C)** PTRR. *P < 0.05, **P < 0.01, ***P < 0.001 and ****P < 0.0001 vs. the CG group (Thermal); ∆ P < 0.05, ∆∆ P < 0.01, ∆∆∆ P < 0.001 and ∆∆∆∆ P < 0.001 vs. the CG group (Mechanical).

Figures 7A,B present the measurements of thermal pain and mechanical pain thresholds for each experimental group at three stages: baseline, after inflammation induction (AII), and after acupuncture treatment (AAT). The measurements of thermal pain and mechanical pain show similar trends. Comparison of pain thresholds before and after inflammation induction shows significant decreases in both thermal pain and mechanical pain across all groups (all P < 0.0001), confirming the successful establishment of the inflammatory pain model. There were no significant differences in thermal pain and mechanical pain between AAT and AII for CG and NR groups. However, when comparing AAT to AII, there were significant increases in pain thresholds in both MA and AR manipulations. Specifically, for thermal pain measurements, both the MA and AR manipulations showed highly significant increases in AAT compared to AII (both P < 0.0001). For mechanical pain measurements, the P values were <0.0001 and <0.001 for MA and AR groups, respectively.

Figure 7C shows the PTRR for thermal pain and mechanical pain measurements across different groups. For thermal pain measurements, comparison between CG and NR indicated that there is no statistically significant difference in PTRR. However, both the MA and AR groups exhibited significant increases in PTRR compared with the CG group, with P values of <0.0001 and <0.01, respectively. There was no significant difference between the MA and AR groups. The PTRR results for mechanical pains were similar to those of thermal pain. These findings suggest that acupuncture by a robotic arm can achieve analgesic effects similar to those of manual acupuncture.

Table 3 shows the data on thermal pain and mechanical pain. Notably, the thermal pain and mechanical pain thresholds of the rats decreased after the initiation of the inflammatory pain model and increased after acupuncture treatment. The thermal PTRR after treatment with NR was 0.10, while the mechanical PTRR was 0.07. In contrast, the thermal and mechanical PTRR after treatment with MA reached 0.34 and 0.43, respectively, indicating a significant improvement. Additionally, the thermal and mechanical PTRR after treatment with AR were 0.35 and 0.45, respectively, suggesting an improved analgesic effect as well.

**TABLE 3:** Thermal and mechanical pain measurement.

Group	Baseline		AII		AAT		PTRR	
	Thermal(s)	Mechanical(g)	Thermal(s)	Mechanical(g)	Thermal(s)	Mechanical(g)	Thermal(s)	Mechanical(g)
CG	13.25 ± 1.84	134.2 ± 18.5	3.38 ± 0.44	42.6 ± 9.08	3.84 ± 0.62	43.9 ± 11.0	0.03 ± 0.05	0.01 ± 0.04
NR	13.14 ± 1.72	115.7 ± 15.7	4.15 ± 0.97	44.9 ± 8.72	5.13 ± 0.60	50.7 ± 6.51	0.10 ± 0.14	0.07 ± 0.11
MA	14.05 ± 1.85	133.4 ± 4.08	3.65 ± 0.43	38.9 ± 4.09	7.11 ± 0.46	79.8 ± 5.41	0.34 ± 0.07	0.43 ± 0.08
AR	13.56 ± 2.20	126.7 ± 9.35	4.37 ± 0.67	36.8 ± 4.26	7.41 ± 0.78	78.0 ± 16.0	0.35 ± 0.21	0.45 ± 0.16

AII: after inflammation induction; AAT: after acupuncture treatment; CG: control group; NR: needle retention group; MA: manual acupuncture group; AR: acupuncture robot group; PTRR: pain threshold recovery rate; Data was presented as mean ± SD.

## 4 Discussion

Acupuncture therapy achieves its analgesic effects by delivering mechanical stimulation to acupoints, triggering a cascade of responses involving connective tissues, cells, molecules, and neural pathways (Zhang et al., 2008). Fundamentally, acupuncture utilizes the mechanosensitive properties of acupoints for therapeutic purposes (Liu et al., 2019). To effectively harness these mechanosensitive properties, understanding how to apply force through acupuncture needles becomes crucial, encompassing key aspects such as force indicators (Deqi assessment), force magnitude, and kinematic parameters. These aspects represent the core elements of acupuncture manipulation techniques, leading to traditionally rigorous training requirements for acupuncturists. However, this traditional approach faces several challenges, including the difficulty and lengthy duration of training, as well as how to preserve the sophisticated manipulation techniques of experienced practitioners (Davis et al., 2012). Several studies have demonstrated that optimal force application techniques and stable manipulation correlate with better therapeutic outcomes (Wang et al., 2023). Clearly, force application and precise, stable needle movement are fundamental to acupuncture manipulation. However, existing acupuncture robots have ignored these critical factors and their therapeutic efficacy has never been evaluated. This study takes the first step towards establishing a robot arm-assisted acupuncture system that can genuinely quantify traditional manual acupuncture and replicate both motion and force parameters during robotic implementation.

In terms of system construction, although joint space information can be reliably executed through the inverse kinematics algorithm of the robotic arm after the trajectory is planned, ensuring precise Cartesian space motion at the robot’s end-effector is crucial for acupuncture procedures. A Laser Vibrometer (LV) was employed to analyze the motion and to evaluate whether the selected robotic arm meets the precision requirements for acupuncture manipulation. According to the Chinese university textbook Techniques of Acupuncture and Moxibustion (Ma et al., 2024), standard LIM parameters typically involve amplitudes of 3–5 mm and frequencies of approximately 1 Hz (60 times per minute). Furthermore, previous research (Tong, 2018) investigating the effects of manual acupuncture on gastric motility demonstrated that frequencies ranging from 1–4 Hz produced varying therapeutic outcomes, with 2 Hz showing optimal effectiveness, while the frequency of 4 Hz showed the least effectiveness. Based on these findings, the validation parameters were designed with more stringent requirements, utilizing frequencies up to 3 Hz and amplitudes below 3 mm, to ensure the robot arm’s capability in executing even more sophisticated acupuncture operations. Evaluation of motion accuracy for robotic arm experiments showed negligible errors at lifting-inserting frequencies below 3 Hz, demonstrating compatibility with manual parameters. The maximum RE was observed for the target motion with a lifting-inserting frequency of 3 Hz and amplitude of 0.5 mm. This suggests that the robot’s precision diminishes under extremely high-frequency and low-amplitude motion planning, potentially reflecting the arm’s trajectory-following characteristics. The MAE increased with the target amplitude, likely due to systematic errors in the motion speed of the robotic arm. These results indicate the robotic arm’s suitability for implementing common clinical acupuncture manipulations, while suggesting that extremely high frequencies and very low amplitudes may be impractical. Subsequently, a real-time needle force monitoring unit was developed with a force sensor mounted on the needle body. Validation experiments conducted on an electronic balance demonstrated that the sensor-measured reactive forces at the needle handle closely matched the pressure transmitted to the balance, with an average absolute error of 0.003N. This indicates that the force measured at the needle handle effectively represents the force applied to the acupoint. Current needle force measurement sensors often suffer from excessive size and weight, with unclear specifications regarding sampling rate and precision. For instance, the “Acusensor” developed by Robert et al. (Oh and Kim, 2022) combines motion and force sensors but is too bulky for manual operation and technique acquisition. The force detection system in Su et al. (2019) acupuncture robot showed a maximum absolute error of 0.155N, risking the loss of crucial force information. The sensor design in this study is compact and lightweight, permitting direct needle installation while facilitating sensitive, real-time force monitoring and recording during acupuncture. Finally, while the LV system provides highly accurate motion data acquisition for both manual acupuncture and robotic replication, its large size limits practical application scenarios. Therefore, an acupuncture manipulation motion acquisition module was developed by applying computer vision technology to enhance operational convenience.

Different acupuncture manipulations exhibit distinct force waveforms and magnitudes (Kwon et al., 2017). In this study, motion and force data were acquired by the system from clinical acupuncturists performing two traditional manual techniques: LIM and Mountain Burning manipulation. These techniques were then replicated by a robotic arm on identical acupuncture models. For both manipulation types, the frequency of robotic motion closely corresponded with the frequency of real-time force signals, confirming the force sensor’s capability for high-speed motion tracking. The robotic replication demonstrated excellent correlation with the quantified manual manipulations, showing high coincidence at multiple characteristic points and achieving accurate reproduction. The slight difference in force between manual and robotic acupuncture, with the robotic method consistently applying slightly less force, may be attributed to factors such as the precision of the robotic system’s force control or differences in the fine adjustments made by human acupuncturists in response to subtle tissue feedback. This minor discrepancy, while noteworthy, does not significantly detract from the overall consistency and accuracy of the robotic replication. These exceptionally high Bhattacharyya coefficients (both close to 1) indicate excellent similarity between robotic and manual operations in both manipulation techniques, demonstrating that the robot can successfully replicate the force characteristics of different manual acupuncture manipulation methods. The concordance between manual and robotic force distributions demonstrates that our robotic system not only replicates the temporal sequence of force application but also maintains the characteristic force patterns of traditional manual manipulation. This statistical similarity in force distribution patterns provides quantitative evidence for the robot’s capability to emulate human-like needling behavior. These findings underscore the capability of robotic systems to accurately replicate the complex motions involved in acupuncture techniques. The high degree of similarity in both motion and force profiles between manual and robotic acupuncture suggests that robotic systems could potentially offer a standardized approach to acupuncture treatment, ensuring consistency across multiple sessions or between different practitioners. This standardization could be particularly valuable in clinical research settings, where controlling variables is crucial for studying the efficacy of acupuncture techniques. Furthermore, the ability of the robotic system to so closely mimic human movements opens up possibilities for preserving and transmitting traditional acupuncture techniques. Expert practitioners’ methods could be accurately recorded and replicated, facilitating both the training of new acupuncturists and the preservation of specialized techniques. In conclusion, the high fidelity of robotic replication in both movement and force application demonstrates the potential of robotic systems in acupuncture.

Ding et al. (2004) developed an intelligent acupuncture robot and conducted human-like acupuncture replication on pigs, though they did not evaluate the therapeutic effects of robotic acupuncture. This study investigated the analgesic effects using the classic complete Freund’s adjuvant arthritis rat model. Among various points along the sciatic nerve, acupuncture at Zusanli (ST36) demonstrated optimal pain relief effects in arthritis rats (Dong and Ma, 2012), leading to its selection for this study. Previous research has shown that the analgesic effect of a 10-min treatment in rats peaked and gradually diminished (Lyu et al., 2019). Based on results, the analgesic effects achieved through robotic acupuncture were comparable to those of manual acupuncture, providing a strong foundation for further development of robotic acupuncture systems.

This study has several limitations. This study did not include investigations on Twisting (TWM), and torque measurements were not reported. The reproduction and evaluation of TWM will be addressed in future studies. While Deqi is a crucial clinical indicator for acupuncture efficacy, kinematic and force analyses were conducted on silica gel simulation skin, which does not exhibit Deqi. Additionally, traditional Chinese acupuncturists rely on tactile force feedback to assess treatment effectiveness, highlighting an area for improvement in robot arm-assisted acupuncture systems with automatic force feedback. The displacement and force characteristics vary across different individuals and acupoints, necessitating extensive research. Future studies should implement the same acupuncture technique on the same acupoint across different individuals using this system. Moreover, it is essential to collect extensive data on the mechanical forces and movement information from animal tissues to create a quantitative dataset for acupuncture techniques. By employing deep learning, models can be trained to understand the relationship between displacement and force at the same acupoint. This might be similar to how practitioners learn to sense Deqi, although Deqi still requires active feedback from the acupuncture recipient, such as soreness, numbness, heaviness, or distension. Ultimately, integrating these findings with large models will facilitate the breakdown of cognitive tasks and enhance human-machine interaction. The effective application of AI technology makes it feasible to develop fully automated intelligent acupuncture robots that combine quantitative analysis with efficacy research.

## 5 Conclusion

This study established a robot-assisted acupuncture system capable of replicating and quantifying manual manipulations with high precision, enabling the objective assessment of motion trajectories and force distributions in acupuncture. The system’s performance was validated through precise force and motion monitoring, demonstrating its ability to standardize acupuncture manipulations. Furthermore, therapeutic efficacy was evaluated using an adjuvant arthritis rat model, providing compelling evidence that robotic acupuncture achieves analgesic effects comparable to manual acupuncture. These findings aim to connect biomechanical signal quantification with physiological effects, addressing a core objective in acupuncture research.

## Data Availability

The original contributions presented in the study are included in the article/Supplementary Material, further inquiries can be directed to the corresponding authors.
